# Chart OR Theme, Depending on Epistemology: Embracing Methodological Shifting

**DOI:** 10.5334/pme.2856

**Published:** 2026-07-08

**Authors:** Lara Varpio

**Affiliations:** 1 Department of Pediatrics, Perelman School of Medicine, University of Pennsylvania, US; 2 Medical Education Collaboratory, The Children’s Hospital of Philadelphia, US

## Abstract

In this commentary, I argue that the injunction to “chart, don’t theme” in scoping reviews needs revision because the methodology has experienced methodological shifting. This means that the, scoping review methodology has evolved because it has moved across epistemological traditions. I contend that charting is methodologically coherent for scoping reviews conducted from an objectivist epistemology, whereas reflexive thematic analysis is coherent for scoping reviews conducted from a subjectivist epistemology. I conclude that researchers should make their epistemological stance explicit and select analytic practices that align with that stance. In summation, I suggest reframing the methodological choice as “chart or theme, depending on epistemology.”

Debate about research methodology is required in health professions education (HPE) for ensuring that a breadth of methodologies are rigorously used in. For that reason, methodological disagreements should be articulated and tested in public scholarly forums, enabling the wider HPE community *to examine and challenge* assumptions and their implications.

In their manuscript “Chart, don’t theme,” Weise, Maggio, and Bennett argue that reflexive thematic analysis (RTA) is not an appropriate analytic approach for scoping reviews. Respectfully, I would like to suggest an amendment to this position. Rather than treating scoping reviews as an unchanging, static methodology, I argue that it is better understood as *evolving*. Methodologies change over time, and so they “exist on a *shifting* continuum” [1, p. 118]; thus, *methodological shifting* is part of a methodology’s history:

“As paradigms, ontologies, and epistemologies change over time, and as methodologies are adopted by different disciplines, methodological traditions shift in accordance with those developments” [1, p. 118].

I propose that scoping review methodology now spans more than one paradigmatic orientation. Depending on whether a researcher works from an objectivist or interpretivist stance, the analytic practices used to enact a scoping review will appropriately differ. Thus, I suggest that the “chart, don’t theme” argument is too categorical. In my opinion, researchers should first identify their epistemic orientation and then, in alignment with it, *chart* ***or*** *theme*.

To develop this argument, I focus on the epistemology and purpose of scoping reviews. Considering each in turn, I argue that RTA and other interpretive analytic approaches can be appropriate in some scoping reviews, just as charting remains appropriate in others. The issue, then, is not whether one practice must displace the other, but how methodological rigor should be calibrated to differing epistemic orientations as the scoping review methodology shifts.

## Scoping Reviews can be Underpinned by Various Epistemologies

I begin with epistemology because the charting versus theming disbute is, at its root, a dispute about what kind of knowledge a scoping review can produce. Scoping reviews can be conducted from within an *objectivist* orientation. As Weise et al correctly observe, when rooted in an objectivist orientation, “[t]he emphasis [of scoping reviews] is on breadth and to show the lay of the land, not to generate new interpretations.” This perspective frames scoping reviews as a form of descriptive synthesis that minimizes interpretation in favor of impartial, transparent mapping. That stance is consistent with an objectivist orientation that frames the scoping review as an as-accurate-as-possible description of the corpus, in which generating “objective knowledge about reality” is the governing ideal [2, p. 545].

More precisely, scoping reviews can be rooted in *relative objectivism*. Weise et al. acknowledge that “all analysis involves some degree of interpretation.” However, as they later note, such interpretation can be restricted by using charting to inform analysis since it “constrains interpretation through predefined categories and operational definitions.” This position follows from a *realist* ontology: reality exists independently of the researcher, even if human access to that reality is always fallible. From this perspective, scoping review should produce the most accurate description of the corpus as possible within the limits of human judgement. When conducted from within such an objectivist frame, the charting practices advocated by Weise et al. are methodologically aligned and, therefore, suited to that aim.

However, I propose that *scoping reviews need not be* ***confined*** *to this epistemological frame*. The descriptive remit of a scoping review can also be understood in subjectivist terms, where description itself is recognized as an interpretive act. In keeping with Thomas et al’s [3] argument that the philosophical stance adopted during a scoping review shapes how information is gathered, analyzed, and interpreted, I suggest that scoping reviews can be conducted from a *subjectivist* epistemology, which holds that:

“individuals construct their own understanding of reality based on interactions with others and with the surroundings (Lincoln and Guba 1985). From this orientation, data and research findings are developed via the interactions between the researcher, the context, and the phenomenon being studied (Crotty 1998)” [3, p. 992].

From this subjectivist orientation, the reviewer is a situated interpreter whose experiences and commitments shape the reading of the corpus. Importantly, Arksey and O’Malley’s 2005 description of scoping reviews makes room for the use of subjective analytic approaches by describe charting as “a technique for **synthesizing and interpreting** qualitative data by sifting, charting and sorting material according to key issues and themes” (emphasis added) [4]. Thus, it is reasonable to argue that scoping reviews can be carried out in a subjectivist orientation since “they bring together the myriad of information on the topic that is available, allowing researchers to offer a **subjective interpretation** of what is known about that topic” (emphasis added) [3, p. 992].

The charting framework created by the researcher(s) itself reflects that subjectivity: it embodies decisions about what deserves extraction, which categories matter, and how findings should be represented. For that reason, when used from a subjectivist orientation, charting does *not* become non-interpretive when categories and operational definitions are specified in advance. The interpretive work occurs in constructing those categories and deciding what counts within them. Predefinition may discipline interpretation, but it does not eliminate it. Therefore, if a researcher undertakes a scoping review from a subjectivist epistemology, RTA is not a methodological error; it is a paradigmatically coherent analytic choice.

If we accept that scoping reviews can be conceptualized within either an objectivist or a subjectivist frame, analytic consequences follow. Charting is especially well suited to objectivist projects; RTA and other interpretive forms of analysis are especially well suited to subjectivist ones. The methodological question is, therefore, not whether scoping reviews are inherently descriptive or interpretive, but which epistemological commitments are being harnessed to guide the review, including analytic approaches.

## Mapping can be Recognized as Having Multiple Purposes

A related issue arises when the purpose of a scoping review is described as cartographic: a scoping review “maps the extent, nature, and distribution of evidence and signals gaps.” That characterization is important, but its implications are shaped by the kind of mapping the reviewer has in mind. If mapping is understood as the objective communication of stable, known information, then charting follows naturally. But that is only one tradition within cartography.

Cartographic scholarship has long challenged the assumption that maps are neutral mirrors of reality. As early as the 1940s, Wright observed that “maps are drawn by men” [5, p. 527] and cautioned that, “[t]he image on a map is drawn by human hands, controlled by operations in a human mind” [5, p. 527]. This insight upholds the contemporary cartography premise that recognizes “the social ‘constructedness’ of maps” and the “power-bound nature of map-making” [6, p. 104]. From this perspective, maps organize landscapes through the assumptions, purposes, and contexts of their makers. That point is as applicable to a corpus of texts as it is to geographic space. A scoping review that “maps” a field need not be objective in the sense imagined by a purely descriptive model. It may equally be understood as a situated construction, shaped by the reviewer’s subjective conceptual and methodological commitments.

The point is not that mapping is always subjective or always objective, but that cartography’s purpose is paradigmatically inflected. Researchers working within an objectivist frame should map the corpus using charting techniques of the kind Weise et al. recommend. Researchers working within a subjectivist frame should map the corpus by adopting interpretivist approaches like RTA. Once again, method should follow epistemological stance.

## Chart OR Theme

If a researcher conceptualizes scoping reviews within an objectivist epistemology, then the advice offered by Weise, Maggio, and Bennett is on point: charting is the more coherent analytic practice, and interpretive analytic approaches like RTA should be avoided.

If, however, a researcher conceptualizes scoping reviews within a subjectivist epistemology, then interpretive analytic approaches like RTA are appropriate means of conducting data analysis.

The broader implication is that scholars need not treat the choice of charting or theming as mutually exclusive methodological selections. I argue that scoping reviews can be conceived of existing on methodologically shifting sands. They need not be framed as fixed procedures. The burden on the researcher is, therefore, to articulate the epistemological stance guiding the review and to ensure that the chosen analytic practices follow coherently from that stance. In that sense, I suggest amending “chart, don’t theme” to *chart or theme, depending on epistemology*.

## Declaration of AI Use

The author would like to acknowledge that Microsoft CoPilot was used in the last stages of manuscript revision. The original draft of the commentary was rather long, but the author was having difficulty finding ways to streamline the argument. Therefore, the author asked CoPilot to: “revise the essay to have a tighter line of argument, while retaining an academic tone, and retaining quotes.” CoPilot did tighten the logic, but it also frequently changed the meaning of the original draft and so the author made several corrections, having failed to appreciate that she could “train” the AI by refining the prompts and having the AI make multiple versions of revisions. Live and learn. As result, the version of the essay submitted for consideration was only approximately 350 words shorter than the original. I note that the revision suggestions from reviewers were much more helpful in reducing the word count. The author assumes full responsibility for the accuracy and integrity of the manuscript, and for the fact that the commentary is likely still too long. In sum, the author would like to acknowledge that the AI support in writing this essay could be labeled as being *somewhat useful*.

## References

[1] Varpio L, Martimianakis MA, Mylopoulos M. Qualitative research methodologies: embracing methodological borrowing, shifting and importing. In: Cleland J, Durning SJ, editors. Researching Medical Education. Chichester, West Sussex: John Wiley & Sons; 2022. pp. 115–125. DOI: 10.1002/9781119839446.ch11

[2] Bergman E, de Feijter J, Frambach J, Godefrooij M, Slootweg I, Stalmeijer R, et al. AM last page: A guide to research paradigms relevant to medical education. Acad Med. 2012;87(4):545. DOI: 10.1097/ACM.0b013e31824fbc8a 22452919

[3] Thomas A, Lubarsky S, Varpio L, Durning SJ, Young ME. Scoping reviews in health professions education: challenges, considerations and lessons learned about epistemology and methodology. Adv Health Sci Educ Theory Pract. 2020;25(4):989–1002. DOI: 10.1007/s10459-019-09932-2 31768787

[4] Arksey H, O’Malley L. Scoping studies: towards a methodological framework. International Journal of Social Research Methodology. 2005;8(1):19–32. DOI: 10.1080/1364557032000119616

[5] Wright JK. Map Makers Are Human: Comments on The Subjective In Maps. The Geographical Review. 1942;32(4):527–544. DOI: 10.2307/209994

[6] Edler D, Kühne O. Deviant Cartographies: A Contribution to Post-critical Cartography. KN – Journal of Cartography and Geographic Information. 2022;72:103–116. DOI: 10.1007/s42489-022-00110-w

